# Characterization of *FMR1* Repeat Expansion and Intragenic Variants by Indirect Sequence Capture

**DOI:** 10.3389/fgene.2021.743230

**Published:** 2021-09-27

**Authors:** Valentina Grosso, Luca Marcolungo, Simone Maestri, Massimiliano Alfano, Denise Lavezzari, Barbara Iadarola, Alessandro Salviati, Barbara Mariotti, Annalisa Botta, Maria Rosaria D’Apice, Giuseppe Novelli, Massimo Delledonne, Marzia Rossato

**Affiliations:** ^1^ Department of Biotechnology, University of Verona, Verona, Italy; ^2^ GENARTIS srl, Verona, Italy; ^3^ Department of Medicine, Section of General Pathology, University of Verona, Verona, Italy; ^4^ Department of Biomedicine and Prevention, Medical Genetics Section, University of Rome "Tor Vergata", Rome, Italy; ^5^ Laboratory of Medical Genetics, Tor Vergata Hospital, Rome, Italy; ^6^ IRCCS Neuromed Mediterranean Neurological Institute, Pozzilli, Italy; ^7^ Department of Pharmacology, School of Medicine, University of Nevada, Reno, NV, United States

**Keywords:** long fragment enrichment, indirect sequence capture, repeat expansion, single nucleotide variants, *FMR1*

## Abstract

Traditional methods for the analysis of repeat expansions, which underlie genetic disorders, such as fragile X syndrome (FXS), lack single-nucleotide resolution in repeat analysis and the ability to characterize causative variants outside the repeat array. These drawbacks can be overcome by long-read and short-read sequencing, respectively. However, the routine application of next-generation sequencing in the clinic requires target enrichment, and none of the available methods allows parallel analysis of long-DNA fragments using both sequencing technologies. In this study, we investigated the use of indirect sequence capture (Xdrop technology) coupled to Nanopore and Illumina sequencing to characterize *FMR1*, the gene responsible of FXS. We achieved the efficient enrichment (> 200×) of large target DNA fragments (~60–80 kbp) encompassing the entire *FMR1* gene. The analysis of Xdrop-enriched samples by Nanopore long-read sequencing allowed the complete characterization of repeat lengths in samples with normal, pre-mutation, and full mutation status (> 1 kbp), and correctly identified repeat interruptions relevant for disease prognosis and transmission. Single-nucleotide variants (SNVs) and small insertions/deletions (indels) could be detected in the same samples by Illumina short-read sequencing, completing the mutational testing through the identification of pathogenic variants within the *FMR1* gene, when no typical CGG repeat expansion is detected. The study successfully demonstrated the parallel analysis of repeat expansions and SNVs/indels in the *FMR1* gene at single-nucleotide resolution by combining Xdrop enrichment with two next-generation sequencing approaches. With the appropriate optimization necessary for the clinical settings, the system could facilitate both the study of genotype–phenotype correlation in FXS and enable a more efficient diagnosis and genetic counseling for patients and their relatives.

## Introduction

The expansion of unstable short tandem repeats is the causal DNA mutation in almost 40 genetic human diseases (Paulson, 2018). This group includes neurological and neuromuscular disorders, such as fragile X syndrome (FXS; MIM# 300624), which is caused by the expansion of CGG trinucleotide repeats in the 5′ untranslated region of the *fragile X mental retardation 1* gene (*FMR1*; MIM# 309550; Eichler et al., 1994; Yrigollen et al., 2012; Nolin et al., 2014). Normal alleles carry 5–44 CGG repeats, whereas expanded alleles are classified as intermediate (45–54 repeats), pre-mutation (55–200 repeats), or full mutation (> 200 repeats). Females with pre-mutations have approximately a 20% risk for fragile X-associated primary ovarian insufficiency (FXPOI; MIM#311360). Older males and females with pre-mutations are at risk for fragile X-associated tremor/ataxia syndrome (FXTAS; MIM#300623). The pre-mutation allele often expands to a full mutation during female germline transmission, thus giving rise to FXS in the progeny. The risk of pre-mutation expansion depends mainly on the number of CGG repeats (with shorter alleles being less likely to expand to a full mutation than larger ones) and the presence of AGG interruptions in the tandem array. Such AGG interruptions increase repeat stability, reduce the risk of expansions (Eichler et al., 1994; Nolin et al., 2003; Yrigollen et al., 2012), and can modulate the disease phenotype (Matsuyama et al., 1999; Sakamoto et al., 2001; Charles et al., 2007; Braida et al., 2010). Moreover, recent evidence has suggested pronounced repeat variability between individuals and within them (mosaicism) that also modulates the disease phenotype (van Blitterswijk et al., 2013; Tabolacci et al., 2020). Similar mechanisms have been observed in the transmission/phenotype of related diseases, such as Myotonic Dystrophy type 1 and Huntington’s disease (Rodriguez and Todd, 2019). Although much less frequent than microsatellite expansions, intragenic single-nucleotide variants (SNVs) and short insertions or deletions (indels) are significant mutational mechanisms leading to FXS and other repeat-associated diseases (Quartier et al., 2017). Accordingly, accurate risk prediction in genetic counseling not only requires the precise characterization of repeats, but also the mapping and counting of interruptions within the repeat array and the ability to map additional intragenic variants (Loomis et al., 2013).

Conventional diagnostic testing to assess repeat length involves triplet repeat primed PCR (TP-PCR) or Southern blotting (Spector et al., 2021). These methods are imprecise when dealing with long expansions, are severely limited in their ability to detect minor alleles, and lack single-nucleotide resolution (Warner et al., 1996; Nolin et al., 2003; Saluto et al., 2005; Filipovic-Sadic et al., 2010; Adler et al., 2011; Bastepe and Xin, 2015; Hayward et al., 2016; Ardui et al., 2018). More recently, third-generation sequencing technologies, such as Oxford Nanopore Technologies (ONT) and PacBio SMRT sequencing, have shown consistent benefits for the characterization of short tandem repeats in FXS and related disorders (McFarland et al., 2014, 2015; Tsai et al., 2017; Giesselmann et al., 2019; Mantere et al., 2019). These approaches can sequence DNA fragments several kbp in length, facilitating the accurate genotyping of repeat expansion alleles and the identification of interruptions and mosaicism (Tsai et al., 2017; Giesselmann et al., 2019). The combination of third-generation sequencing with enrichment strategies can reduce costs while ensuring sufficient coverage for accurate repeat characterization by focusing on the target site. In the first such report, *FMR1* repeat arrays were amplified by PCR for PacBio sequencing (Loomis et al., 2013). However, PCR is unsuitable in patients heterozygous for normal and large expansion alleles because only the normal allele may be amplified (Chakraborty et al., 2016), and polymorphisms surrounding the repeat region can lead to allele bias, dropout, or the misinterpretation of results (Bastepe and Xin, 2015).

More recently, both third-generation sequencing technologies have been coupled to an enrichment method based on CRISPR/Cas9, where Cas9 cuts at sites flanking the repeats allowing the ligation of sequencing adapters for the accurate characterization of repeat length, interruptions, and mosaicism in *FMR1* (Tsai et al., 2017). Although this removes the reliance on PCR, remaining limitations include the large amount of starting material required, typically 1–10μg DNA (Gilpatrick et al., 2020; Stangl et al., 2020), which makes it difficult to work with low-abundant samples, as, for examples, those from prenatal/pre-implant testing or clinical biopsies. Moreover, sequencing is confined to a few kbp surrounding the repeat, thus preventing the analysis of mutations along the full length of the causative gene. Finally, the system lacks flexibility, because commonly utilized protocols to sequence the Cas9-enriched DNA rely only on long-read sequencing and not on short-read sequencing platforms, such as Illumina, which show higher accuracy. Despite recent improvements strongly increased long-read accuracy, ONT still fails at accurately detecting indels (Maestri et al., 2020), while PacBio High-Fidelity mode still requires the use of high-capacity SMRT cells, that makes the analysis very expensive when only few samples are multiplexed. These are critical drawbacks, especially when the repeat characterization is inconclusive and the analysis of the entire gene is necessary to identify other mutations, namely, SNVs or indels (Sitzmann et al., 2018). Although the analysis of tandem repeats using Illumina technology is challenging due to the large size and typically high GC content of the fragments, it has nevertheless proven valuable for the identification of causative intragenic variants in patients with a negative standard workup based on the analysis of repeat expansions (Quartier et al., 2017). To address these limitations and exploit the advantages of both short-read and long-read sequencing, we investigated the use of Xdrop technology (Samplix, Birkerød, Denmark) for the characterization of the *FMR1* locus. The approach uses so-called “indirect sequence capture” to enrich for long fragments (several kbp) starting with limited DNA input (10–15ng). High-molecular-weight (HMW) DNA molecules (50–100 kbp) are initially encapsulated in individual droplets, and droplet PCR (dPCR) is used to amplify a detection sequence (DS) of 100–150bp located near the target of interest. Positive droplets are revealed by staining with a DNA-intercalating dye and are recovered by flow sorting. A few hundred target DNA molecules are recovered for multiple displacement amplification after their encapsulation in individual droplets (dMDA) to minimize amplification biases (Madsen et al., 2020; Blondal et al., 2021). We took advantage of Xdrop technology to enrich the *FMR1* locus and used ONT long-read sequencing to characterize the *FMR1* repeat length/features with parallel Illumina sequencing to determine the presence of intragenic variants within the *FMR1* gene body.

## Materials and Methods

### DNA Samples

Genomic DNA (NA12878, NA06891, NA07537, and NA20241, representing cells with diverse *FMR1* alleles) was purchased from the Coriell Institute for Medical Research. All the other samples were isolated from the whole blood of unrelated healthy donors (Blood Center, Verona Hospital) following informed written consent. Venous blood samples were collected in EDTA tubes, de-identified immediately after collection, and stored at −80°C until use. The study was approved by the Ethics Committee for Clinical Research of Verona and Rovigo Provinces and all the investigations were conducted according to the Declaration of Helsinki. Genomic DNA was extracted using the Genomic Tip 100/G kit (Qiagen, Hilden, Germany), Nanobind CBB Big DNA Kit (Circulomics, Baltimore, MD, United States), NucleoSpin Blood Mini kit (Macherey-Nagel, Düren, Germany), or the Miller’s protocol (Miller et al., 1988). All protocols were carried out according to the manufacturer’s instructions, and for the Circulomics kit, we used either the HMW or ultra-HMW protocol. The different DNA extraction methods were tested on samples from distinct donors, an aspect that may represent a weakness of the study.

### Droplet Generation and dPCR

Before enrichment, DNA samples were purified using 1× HighPrep MagBio beads (MagBio Genomics, Gaithersburg, MD, United States) and diluted with DNase-free water to 5ng/μl. Detection sequence-specific primers for *FMR1* enrichment were designed using the Samplix primer design tool^1^: forward primer 5'-GAG CCC TAG TCC TCA CCC AAT-3' and reverse primer 5'-CCC TAC CTA TCA GGC AAA GCT-3' (Supplementary Figure S1). The dPCR reaction consisted of 20μl 2× dPCR mix (Samplix), 0.8μl of each primer (10μM), 2μl 5ng/μl DNA, and water to 40μl. Droplets were generated using a dPCR cartridge and Xdrop droplet generator (both from Samplix). Droplets were then transferred to four tubes and dPCR was carried out by heating to 94°C for 2min followed by 40cycles of 94°C for 3s and 60°C for 30s at a ramping rate of 1.5°C/s.

### Positive Droplet Sorting

Following dPCR, droplets were collected in a single tube, diluted with 1ml dPCR buffer (Samplix), and stained with 10μl droplet dye (Samplix). Droplets were sorted on a FACS Aria Fusion II (Becton Dickinson, Franklin Lakes, NJ, United States), with instrument settings adjusted to FSC=210, SSC=250, and FL1=370. The positive droplets were gated on FL1 fluorescence and the sorting mode was set to “Yield.” Sorted droplets were collected in 15μl water.

### dMDA

Sorted droplets were mixed with 20μl Break solution and 2μl Break color (Samplix), and 10μl of the resulting aqueous phase was used as a template for dMDA. The reaction mix consisted of 4μl dMDA buffer, 1μl dMDA enzyme, 10μl template, and water to 20μl. Droplets were generated as above, while running the dMDA program. Afterward, the droplets were incubated for 16h at 30°C (lid at 75°C) followed by 10min at 65°C to terminate the reaction. The dMDA droplets were broken using 20μl Break solution and 1μl Break color as above.

### qPCR Analysis

Total DNA released from dMDA droplets was quantified using a Qubit fluorimeter and the Qubit HS DNA quantification kit (Thermo Fisher Scientific, Waltham, MA, United States). The size range of the amplified DNA was analyzed on a TapeStation 4,150 using the Genomic DNA ScreenTape assay (both from Agilent Technologies, Santa Clara, CA, United States). Fold enrichment of target DNA was assessed by qPCR using the KAPA library Quant qPCR mix (Roche, Basel, Switzerland), 10ng DNA, and 2mM each of forward (5′-TCA TTG GTG GTC GGG TGT AC-3′) and reverse (5′-AGC GAC ACC TCA CAT TCC TT-3′) validation primers (Supplementary Figure S1). Fold enrichment was determined using an online calculator.^2^ Usually, samples with ≥100-fold enrichment at qPCR showed also robust enrichment and breath of coverage after sequencing and thus were selected for downstream analysis.

### ONT Sequencing

We sequenced 1–1.5μg of the enriched DNA samples from the Xdrop workflow using the ONT platform, pooling two replicates when necessary. Amplified DNA was initially debranched using 15units of T7 endonuclease I in 30μl for 15min. Debranched DNA fragments were isolated by size selection using AmPure XP beads (Beckman Coulter, High Wycombe, United Kingdom) in the presence of 15% polyethylene glycol (Sigma–Aldrich, St Louis, MO, United States). The ONT sequencing library was generated using the Oxford Nanopore Ligation Sequencing Kit SQK-LSK109 (ONT, Oxford, United Kingdom) according to the manufacturer’s instructions with minor modifications. Briefly, DNA was end-repaired using the NEBNext FFPE DNA Repair Mix (New England Biolabs, Ipswich, MA, United States) at 20°C for 10min and subsequently end-prepped with the NEBNext End repair/dA-tailing Module (New England Biolabs) at 20°C for 20min. Sequencing adapters were ligated at room temperature for 10min. Finally, the 30–50 fmol library was loaded into a MinION R9.4.1 flowcell (ONT) and standard settings were applied for a run time of ~16h.

### ONT Data and Repeat Analysis

Base calling was applied to the raw ONT fast5 files using Guppy v4.2.2 in high-accuracy mode, with parameters “-r -i $FAST5_DIR -s $BASECALLING_DIR --flowcell FLO-MIN106 --kit SQK-LSK109.” Reads were quality filtered using NanoFilt v2.7.1 (De Coster et al., 2018), with a minimum quality score of 7. Reads were then mapped to the hg38 human reference genome using Minimap2 v2.17-r941 (Li, 2018). The ONT datasets showed a large fraction of bases (59.3%) mapping as supplementary alignments within the same genomic region, but not recurrent at the same position, suggesting the presence of chimeric reads, possibly derived from dMDA as previously reported (Gawad et al., 2016; Zhou et al., 2020). To exploit the full sequencing dataset, ONT read mapping was therefore adjusted by also considering supplementary read alignments. Bedtools intersect v2.29.2 (Quinlan and Hall, 2010) was used to extract primary or supplementary alignments completely spanning the *FMR1* repetitive region defined in a bed file, containing repeat coordinates plus 400bp flanking the repeat on each side (chrX:147911849–147,912,310). Sequences corresponding to alignments of interest were extracted in forward orientation from the bam alignment file using a combination of Samtools v1.10 (Li et al., 2009) and awk scripting language and were realigned to the hg38 reference using Minimap2. A combination of PcrClipReads and SamExtractClip from jvarkit v1f97a3401^3^ and seqtk subseq v1.3-r106^4^ was then used to trim the portions of sequences outside the bed file, allowing us to retrieve all sequences fully spanning the repeat, including supplementary alignments.

Repeat length was determined from consensus sequences obtained by the *de novo* assembly of the extracted sequences using the *CharONT* pipeline (Supplementary Figure S2). First, the sequences were clustered using VSEARCH v2.15.1_linux_x86_64 (Rognes et al., 2016) with an 85% minimum identity threshold. Reads in the most abundant cluster were then aligned to each other using MAFFT v7.475 (Katoh et al., 2002) with parameters “--auto –adjustdirectionaccurately.” A draft consensus sequence was called using EMBOSS cons v6.6.6.0,^5^ setting the “--plurality” parameter to the value obtained by multiplying the number of aligned reads by 0.15 (Maestri et al., 2019). This process generated a preliminary consensus sequence for one allele. All sequences were then mapped to the consensus sequence, and a bidimensional score was calculated for each sequence, extracting the size of the biggest DEL, and the biggest INS from the CIGAR string in the bam file. If soft clipping occurred, the length of the soft-clipped sequence contributed to the score calculation by exploiting the presence of flanking sequences. Candidate outliers were then identified (with either component of the score exceeding a predefined threshold based on the interquartile range of scores assigned to all sequences) and were excluded from the clustering process. Scores were used to cluster the sequences in two groups, corresponding to the two alleles, using the *k*-means function of the “stats” R package (R Core Team, 2013). Outliers with either component of the score exceeding a predefined threshold were then identified based on the interquartile range of scores assigned to sequences within the cluster and were saved to a new file. Sequences assigned to each allele were processed separately. Up to 200 sequences were randomly subsampled using seqtk sample, and a draft consensus sequence was called by combining MAFFT and EMBOSS cons, as previously described (Footnote 5). Another set of up to 200 reads was subsampled using seqtk sample to polish the draft consensus sequence, and read overlaps were found with Minimap2 (Li, 2018). Racon v1.4.13 (Vaser et al., 2017) was then used to perform a first round of polishing with parameters “-m 8 -x−6 -g−8 -w 500 --no-trimming.” A second round of polishing was performed using the medaka_consensus program of Medaka v1.2.1^6^ specifying the “r941_min_high_g360” model. The polished consensus sequences for each allele were finally searched for repeat motifs using Tandem Repeat Finder v4.09 (Benson, 1999). The scripts used to generate consensus sequences and repeat annotations are available online.^7^

The presence of somatic mosaicism was investigated by aligning reads to sequences flanking the repeat, searching for repeat motifs, and visualizing alignments in a genome browser using the *MosaicViewer_FMR1* pipeline (Supplementary Figure S3). The msa.sh and cutprimers.sh programs from BBMap suite v38.87 were used to trim one of the two sequences flanking the repeat expansion, and trimmed reads were aligned to the other flanking sequence using Minimap2. Alignments were visualized in the IGV genome browser v2.8.3 (Robinson et al., 2011). Mapped sequences were extracted from the bam file in the forward orientation using Samtools and a custom script, and the ID of reads in reverse orientation was extracted from the SAM flag. Extracted sequences were searched for repeats with the motif “CGG” using the NCRF script in the Noise-cancelling repeat finder package v1.01.02 (Harris et al., 2019) with parameters “--scoring=nanopore --minlength=12 CGG_repeat:CGG --minmratio=0.90 --stats=events –positionalevents.” Repeats were sorted in a single repeat summary file using the scripts ncrf_cat.py, ncrf_sort.py, and ncrf_summary.py. Reads were then aligned to the flanking sequence using Minimap2 and visualized in the IGV genome browser. The scripts used to investigate somatic mosaicism are available online.^8^

### Illumina Sequencing

Amplified DNA was fragmented using a Covaris sonicator to achieve an average size of 400bp, and Illumina PCR-free libraries were prepared from ~200–400ng DNA using the KAPA Hyper prep kit and unique dual-indexed adapters (5μl of a 15μm stock) according to the supplier’s protocol (Roche). The library concentration and size distribution were assessed on a Bioanalyzer (Agilent Technologies). Barcoded libraries were pooled at equimolar concentrations and sequenced on a NovaSeq6000 instrument (Illumina, San Diego, CA, United States) to generate 150-bp paired-end reads.

### Illumina Data Analysis and Variant Calling

Illumina fastq files were quality checked using FastQC,^9^ and low-quality nucleotides and adaptors were trimmed using fastp (Chen et al., 2018). Reads were then aligned to the reference human genome version GRCh38/hg38 using BWA-MEM v0.7.17.^10^ All bam files were cleaned by local realignment around indel sites, followed by duplicate marking and recalibration using Genome Analysis Toolkit v3.8.1.6. BamUtil v1.4.14 was used to clip overlapping regions of the bam file in order to avoid counting multiple reads representing the same fragment. The genotypability of the *FMR1* gene was calculated using CallableLoci in GATK v3.8, with a minimum read depth of 10. CollectHsMetrics by Picard v2.17.10 was used to calculate fold enrichment to determine enrichment quality. Variants were called using HaplotypeCaller (GATK v4.1.8.0). Variant filtering was then carried out according to the GATK Best Practices for exomes. Variants were also filtered by quality (filter PASS) and by location within the *FMR1* gene. The accuracy of variant calling for each replicate was calculated using SNPSift, comparing their genotypes with the GIAB NA12878_HG001 annotated VCF file,^11^ based on variants called by at least two different pipelines. Variants were annotated using VarSeq (GoldenHelix, Bozeman, MT, United States) to screen clinical databases of germline mutations: ClinVar and HGMD Professional v2020.1.

## Results

### *FMR1* Enrichment Using Xdrop Technology

A specific primer pair was designed to amplify a DS by dPCR ~5 kbp from the microsatellite repeat in exon 1 of the *FMR1* gene (Supplementary Figure S1). Another primer pair was designed to anneal ~500bp from the latter in order to monitor enrichment by qPCR (Supplementary Figure S1).

**Figure 1:**
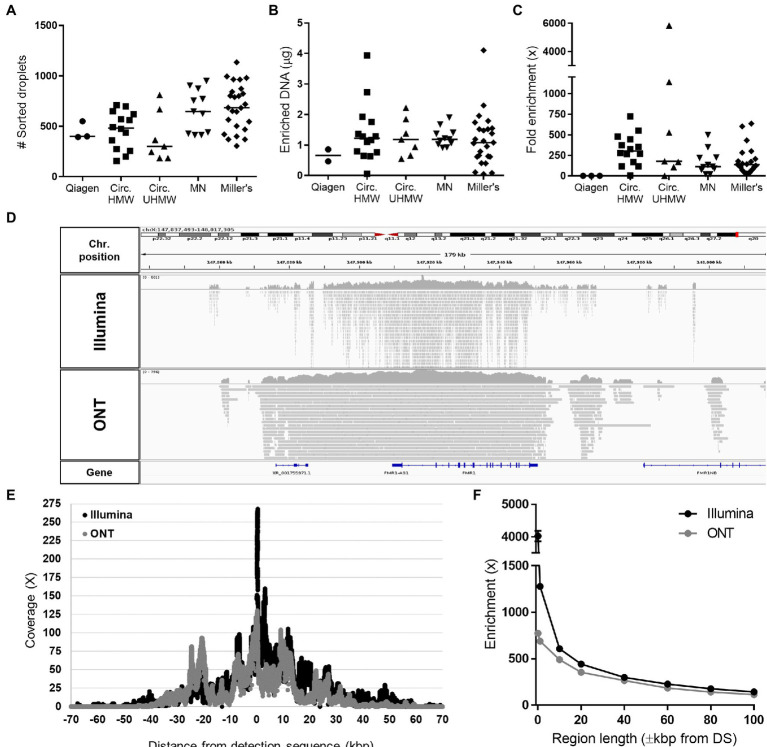
Statistics of *FMR1* enrichment using the Xdrop technology. **(A)** Number of positive sorted droplets, **(B)** quantity of amplified DNA recovered after dMDA, and **(C)** fold enrichment of *FMR1* determined by qPCR after applying the Xdrop workflow to DNA samples extracted with different methods: Genomic Tip kit (Qiagen), Circulomics Nanobind CBB Big DNA Kit using either the HMW protocol (Circ. HMW) or the ultra-HMW protocol (Circ. UHMW), the NucleoSpin Blood Mini kit (Macherey-Nagel, MN), or Miller’s protocol (Coriell samples). **(D)** Integrative Genomics Viewer (IGV) visualization of Illumina and ONT mapped reads obtained from a representative Xdrop-enriched sample. **(E)** Average coverage and **(F)** fold enrichment of the *FMR1* gene after sequencing the Xdrop-enriched samples listed in Supplementary Table S1 on the ONT and Illumina platforms.

The Xdrop *FMR1* assay was tested on samples comprising DNA fragments >60 kbp extracted using five different methods (Supplementary Figure S4A). Following Xdrop-mediated encapsulation and dPCR, a clear cloud of positive droplets was visible by FACS for all but one of the extraction methods (Supplementary Figure S4B). We sorted an average of ~500 positive droplets for each sample, allowing the recovery of ~1.3μg of enriched DNA after dMDA (Figures 1A,B), each of which was 12–15 kbp in length (Supplementary Figure S4C). The *FMR1* target showed a median enrichment of 170× across all samples based on qPCR analysis (Figure 1C). Although the Circulomics ultra-HMW protocol resulted in highly variable enrichments, no significant differences were observed among the extraction methods on average, with the exception of Qiagen columns (which did not achieve successful enrichment).

A subset of Xdrop-enriched DNA samples was sequenced using the Illumina and ONT platforms, generating on average 11,493,290 and 170,532 reads, with average lengths of 150 and 4,098bp, respectively (Supplementary Table S1). Both sequencing methods achieved low genome-wide coverage (~0.2×) but significant enrichment was reproducibly observed for all samples on the *FMR1* gene: 462× for Illumina and 357× for ONT (Figure 1D; Supplementary Table S1). Maximum enrichment for both sequencing technologies was observed on the DS, and progressively decreased moving away from the target site, with a coverage >10× maintained for up to ±40 kbp flanking the DS (Figures 1E,F).

### Analysis of *FMR1* Repeat Characteristics by Xdrop Enrichment and ONT Sequencing

**Figure 2:**
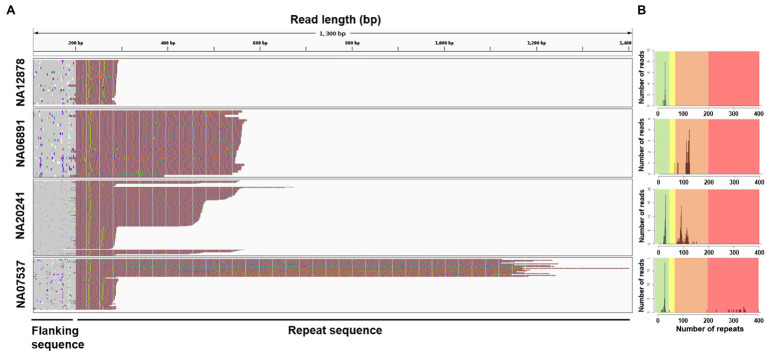
Visualization of repeat structure and length after sequencing Xdrop-enriched samples on the ONT platform. **(A)** Individual ONT reads were trimmed to include only the *FMR1* repeat region plus 400bp flanking sequence and aligned at the repeat 5'-end. Each line represents a single read, colored according to: A=green, T=red, G=orange, and C=blue. **(B)** Repeat count histograms showing the number of reads reporting a certain repeat length: shaded background in each plot represents risk ranges for disease development. Green=normal; yellow=intermediate; orange=pre-mutation; and red=full mutation.

Next, we analyzed ONT sequencing data representing samples with known repeat features and showing expansions of 100–1,000bp (Table 1). The consistent enrichment achieved on the target (range 33–330×) facilitated the extraction of sufficient reads spanning the entire tandem array (22 to 257) and allowed us to determine allele counts and features for every sample (Figure 2 and Table 1). Sample NA12878 showed the anticipated normal pattern of 28 CGG repeats in both alleles, interrupted by the AGG trinucleotide at two sites. Sample NA06891 was derived from a male patient in the pre-mutation stage, with 118–121 CGG repeats according to previous sequencing data (Amos Wilson et al., 2008; Lim et al., 2017). Consistently, our analysis counted an average of 119 CGG repeats and highlighted the presence of a single AGG trinucleotide interrupting the array. Sample NA20241 was obtained from a female patient heterozygous for normal and pre-mutated alleles. The expanded allele was reported to contain 93–110 repeats based on traditional methods (Amos Wilson et al., 2008), whereas more recent PacBio sequencing analysis revealed two groups of molecules with 90 and 120 repeats, respectively (Tsai et al., 2017). In agreement with the latter study, our analysis demonstrated the presence of mosaicism in this sample, evident as a bimodal distribution of sequencing read lengths, with modal values of 92 and 113 repeats. The CGG repeat count of the normal allele was also confirmed as 29, interrupted by two AGG trinucleotides. Sample NA07537 was previously reported to be heterozygous with 29 CGG repeats in the normal allele and>200 in the expanded allele, corresponding to a full mutation (Adler et al., 2011). The expanded allele was also characterized by PacBio sequencing, revealing a broad size distribution of 272–400 CGG repeats, which was confirmed by our data. Specifically, ONT sequencing reads ranged from a minimum of 196 to a maximum of 402 repeats, with a modal value of 342. Overall, the analysis of Xdrop-enriched samples by ONT sequencing allowed the accurate assessment of *FMR1* repeat length for each allele, and their correct classification as normal, pre-mutation, or full mutation. Moreover, the per-base analysis revealed repeat interruptions and mosaicism in agreement with previous reports.

**Table 1:** Characterization of *FMR1* repeats from Xdrop-enriched samples by ONT sequencing.

Sample ID	Condition	Sex	Total ONT reads	Mean coverage on repeat	Fold enrichment on repeat	Reads spanning repeat	Allele	Average number of expected repeats	Average number of observed repeats
NA12878	Normal	F	96,549	33.0X	713.9	22	1	28 CGG+2 AGG	28 CGG+2 AGG
							2	28 CGG+2 AGG	28 CGG+2 AGG
NA06891	Pre-mutation	M	265,556	55.5X	791.2	36	1	118 (Amos Wilson et al., 2008)	119 CGG+1 AGG
								121 (Lim et al., 2017)	
							2	–	–
NA20241	Pre-mutation	F	94,262	330.8X	930.3	257	1	29(Chen et al. 2010; Juusola et al. 2012; Lim et al., 2017)	27 CGG+2 AGG
								27CGG+2AGG (Tsai et al., 2017)	
							2	93–110(Amos Wilson et al., 2008)	114 CGG+1 AGG (mosaicism)
								125(Lim et al., 2017)	
								119, mosaicism (Tsai et al., 2017)	
NA07537	Mutation	F	737,359	146.8X	912.9	87	1	28–29 (Adler et al., 2011)	27 CGG +2 AGG
								27 CGG+2 AGG (Tsai et al., 2017)	
							2	>200, mosaicism (Adler et al., 2011; Lim et al., 2017; Tsai et al., 2017)	342 CGG+1 AGG (mosaicism)

*For each sample, ONT sequencing statistics covering the FMR1 repeat are shown, along with the anticipated repeat features based on earlier reports and data generated from Xdrop-enriched samples*.

### Analysis of *FMR1* Intragenic Variants by Xdrop Enrichment and Illumina Sequencing

Xdrop allowed the enrichment of a genomic region containing the entire *FMR1* gene, so we next analyzed intragenic SNVs and indels in the same four samples discussed above (Supplementary Table S1). At this aim, we exploited Illumina sequencing (Supplementary Table S1), because with ONT most of gene body (74%) had coverage <60X (Figure 1F), i.e., lower than the minimum threshold required to accurately call SNV using this technology (Maestri et al., 2020). Analysis of the five distinct dMDA replicates of the NA12878 sample, for which genotypes are available, demonstrated most of the GIAB variants were properly called by each replicate, with 93% sensitivity on average (Supplementary Table S2). A minor fraction of false-positive (FP, 9%) and false-negative (FN, 7%) variants was also identified, but not reproducibly detected among replicates. FN variants were caused by non-callable/non-covered positions or allele dropout, whereas FP variants were usually supported by low read depth (<15 reads) and characterized by low Variant Allele Frequency (VAF<25%, in 67% of the FP cases).

Based on these results, to avoid FNs, variants were called on the other three *FMR1* cases considering both available dMDA replicates (Table 2). Each sample showed an average coverage breadth >5× and genotypability ranging from 91 to 100% on *FMR1*. The consideration of both replicates allowed the entire *FMR1* gene length to be genotyped (99.99%), including the 34 positions of pathogenic/likely pathogenic variants listed in clinical databases (Table 2 and Supplementary Table S3). These positions could be genotyped in all samples by both replicates, except for two variants in sample NA06891 that could be called based on only a single replicate (Table 2 and Supplementary Table S3). No variant was identified in these positions, in agreement with the absence of pathogenic SNVs/indels reported within the *FMR1* gene for these samples (Table 2). These results confirmed that Xdrop enrichment coupled to Illumina sequencing allows the analysis of clinically relevant variants in the *FMR1* gene, but the use of technical dMDA replicates is necessary for complete, high-confidence variant calling.

**Table 2:** Genotyping data for the *FMR1* gene from Xdrop-enriched samples based on Illumina sequencing.

Sample ID	Replicate	Average coverage FMR1	%5x	%10x	%PASS	%PASS DP10	Total %PASS	Number of variants identified	Pathogenic variants	Reported pathogenic variants genotypable
NA12878	R1	293	100	98.74	98.99	97.17	100	20	0	34/34
	R2	244	99.58	95.54	96.44	91.41		22		
	R3	707	95.58	93.01	93.61	91.1		18		
	R4	52	95.05	85.62	96.89	84.04		22		
	R5	114	100	99.37	100	97.58		20		
NA06891	R1	257	91.3	90.4	91.3	90.2	99.9	35	0	34/34
	R2	164	100	100	99.9	99.9		52		
NA20241	R1	544	100	100	100	100	100	37	0	34/34
	R2	873	100	100	100	100		38		
NA07537	R1	336	98.3	96.6	98	96.2	100	42	0	34/34
	R2	400	100	99.3	100	99.2		41		

*For each replicate, columns show the average coverage of Illumina data on FMR1, the percentage of the FMR1 gene covered by at least 5 or 10 reads, the percentage of callable bases on the target for standard read depth (> 3, %PASS) or read depth>10 (%PASS DP10), and the total number of variants identified, considering the combination of replicates, the total percentage of callable bases on the target for read depth>3, the pathogenic variants identified, and the fraction of known pathogenic/likely pathogenic variants in PASS positions*.

## Discussion

The accurate characterization of short tandem repeats, which underlie numerous inherited diseases, is challenging to achieve using traditional methods and even with the most recent sequencing technologies. Yet the correct diagnosis of these diseases and informed prognosis requires the precise determination of the number of repeats as well as the complete and accurate characterization at single-nucleotide resolution of both the repetitive site and the surrounding regions.

In this study, we demonstrated that the size and composition of triplet repeats in the *FMR1* gene can be determined accurately by Xdrop enrichment coupled to ONT long-read sequencing. The approach allowed us to classify the full range of *FMR1* alleles (normal, pre-mutation, and full mutation), with accurate size estimates comparable to previous results. Furthermore, the enrichment of sequencing data at the target site was sufficient to compensate for the consistent frequency of ONT sequencing errors, thus allowing the high-confidence identification of AGG interruptions. This aspect is essential because the presence of one or zero interruptions within a pre-mutated allele confers a high risk of expansion into a full mutation (Nolin et al., 2003; Yrigollen et al., 2012). In FXPOI patients, presence of AGG interruptions has also an effect on the fragile X-associated ovarian dysfunction (Lekovich et al., 2018). The precise determination of interruption patterns in female (pre-mutation) carriers is therefore critical because it influences their reproductive planning. Depending on the expansion risk, women might opt for preimplantation genetic diagnosis or normal conception, optionally combined with invasive prenatal diagnosis to screen the fragile X status of their fetus (Coskun and Alsmadi, 2007; Chen et al., 2020). In this context, Xdrop technology offers advantages over the Cas9 approach, because 500–1,000 times less DNA is required, allowing the application of long-read sequencing to limited samples, such as those derived from prenatal testing (Mosca-Boidron et al., 2013). In addition, the inclusion of an additional dMDA step before the conventional Xdrop workflow may enable target enrichment even from a single cell, as required for preimplantation testing (Hård et al., 2021).

In addition to repeat interruptions, we also detected a consistent level of mosaicism affecting the size of tandem repeats in pre-mutated and fully mutated alleles. Repeat instability is a hallmark of repeat expansion disorders (Nolin et al., 1999), and it may also explain why the accurate sizing of repeats in previous studies using traditional methods has been so challenging (Tsai et al., 2017). Assessing the variability in CGG repeats within and between tissues is another important aspect of FXS and FXTAS diagnosis because this can influence the clinical phenotype of affected individuals (Pretto et al., 2014). Although our results confirmed the presence of somatic mosaicism, in agreement with previous reports based on long-read sequencing (Tsai et al., 2017; Hafford-Tear et al., 2019; Sone et al., 2019), we also observed some variability within clusters, which may reflect the accumulation of indel errors along ONT reads.

From the technical perspective, the level of enrichment achieved with the Xdrop technology, typically >200x, was comparable to other targeted enrichment strategies associable to long-read sequencing (Tsai et al., 2017; Gilpatrick et al., 2020). Also, the genome-wide noise of Xdrop samples (˜0.21X) was similar to that obtained with the Cas9 system coupled to ONT (˜0.3X) in our hands. Currently, the workflow for Xdrop-based enrichment takes ˜1.5days and ˜150 € per sample, both anticipated to be reduced with the expected release of a Xdrop system integrated with a flow sorter. After enrichment, sequencing costs are comparable for the Xdrop- and the Cas9-system, that are both compatible with ONT and PacBio long-read technologies, and Xdrop also with Illumina. In our experiments, the high enrichment achieved using Xdrop technology facilitated downstream analysis with a limited amount of sequencing data (< 1 million ONT reads and~10 million Illumina reads). The Xdrop workflow also provides an option to assess enrichment by qPCR before proceeding with sequencing. This should be considered solely as a qualitative test to ensure successful results (when >100x), because there was no full correlation between the enrichment level determined by sequencing and qPCR in our experiments. One potential drawback is the generation of chimeric reads, despite the robust limitation of this phenomenon by droplet-based Phi29 amplification (Zhou et al., 2020; Gonzalez-Pena et al., 2021; Hård et al., 2021). Chimeras can be overcome by considering supplementary alignments, even if such adjustments reduce the length of ONT read mapping, thus limiting the ability to accurately assess repeat lengths expanding over primary alignment sizes. This issue could be addressed by reducing the dMDA reaction time or using more efficient sorting systems. The breadth of enrichment achieved using Xdrop technology spanned a~60–80 kbp region flanking a single “point of view” (DS) sequence of 100bp, and probably corresponded to the average length of the initial DNA molecules encapsulated in the droplets. This is an advantageous feature because it allows the analysis of the entire *FMR1* gene, far beyond the triplet repeat stretch. This option is not readily available when using the Cas9 enrichment approach, which is typically limited to the distance specified by the guide RNA location (≤ 20 kbp). To maximize the breadth of coverage covering the whole gene, HMW starting DNA is preferred, but ultra-HMW molecules should be avoided because viscous DNA is difficult to dilute down to nanograms with any accuracy, resulting in variable enrichments (as we experienced with the Circulomics ultra-HMW protocol). In our hands, a wide set of DNA extraction methods provided similar enrichment results, even when not specifically designed for HMW DNA extraction (i.e., MN, based on standard silica columns). The possibility to use extraction kits routinely used in diagnostic procedures as well as frozen blood, as our starting samples could facilitate the broad application of the technology in the clinic. An exception was the Qiagen Gtip kit that did not properly work in combination with Xdrop. The latter may reflect the carryover of contaminants that interfere with DNA encapsulation/staining and could not be removed using bead-based cleanup methods.

Investigation of the full FMR1 gene is beneficial when the analysis of repeat expansion is inconclusive and the exclusion of other mutations within the gene body is desirable either to complete genetic testing or to prevent disease transmission. Indeed, besides repeat expansion, FXS can be also caused by point mutations or deletions, as those recently reported to occur in the 5’UTR of FMR1, that can challenge genetic diagnosis (Erbs et al., 2021). Although rare, these variants could be fine characterized at the breakpoint level by the Xdrop method, thus facilitating the segregation analysis and genetic counseling. We genotyped SNVs along the entire *FMR1* gene by coupling Xdrop enrichment to Illumina sequencing. Because the dMDA step yields sufficient DNA for both protocols, the same Xdrop-enriched DNA can therefore be sequenced on the ONT platform for the analysis of repeat expansions, and then with Illumina technology to assess the presence of intragenic variants. Based on this approach, we could accurately genotype the entire *FMR1* gene and analyze all pathogenic/likely pathogenic variants reported therein. The small fraction of FP and FN calls was not reproducible in multiple samples and could therefore be identified by analyzing two sequencing replicates from different dMDA reactions. Accordingly, the same initial sorted sample could be split in two aliquots for independent downstream amplifications. Because FN calls were mainly due to coverage drops, these could be minimized by adding a second DS in the same reaction, to allow more uniform enrichment over the 80 kbp length of the gene, especially when using not-HMW DNA. FP calls were probably caused by dMDA errors, because the constant amplification of a few hundred molecules obtained by sorting may result in this frequency of artifacts, as well documented for single-cell sequencing (Gawad et al., 2016). We excluded the possibility that FP calls were derived from contamination prior to the dMDA step, such as sorting processing or the operator, because negative controls (sequencing the sheath fluid from the flow sorter) did not reveal the presence of human DNA. Preliminary data also suggested that FP calls may be exacerbated when the efficiency of sorting is suboptimal because this reduces the number of target molecules collected and thus increases the chance of Phi29 errors. The anticipated launch of an Xdrop system integrated with a flow sorter should maximize the efficiency of this method, thus overcoming such technical limitations.

## Conclusion

Our study demonstrated the simultaneous characterization of challenging microsatellite expansions and SNV/indels within the *FMR1* gene, which has not been achieved before. This was possible thanks to the implementation of a novel targeted sequencing approach, in which Xdrop enrichment was combined with the analysis of large DNA fragments by short-read and long-read sequencing. Although technical improvements are required to implement this approach in the clinic, our proof-of-concept study should be easily adapted for the analysis of other genes characterized by repeat expansions, or other genomic loci where the analysis of structural variations combined with the detection of SNVs and indels is desirable for complete genetic counseling.

## Data Availability Statement

The datasets presented in this study can be found in online repositories. The names of the repository/repositories and accession number(s) can be found at: https://www.ncbi.nlm.nih.gov/, PRJNA745542.

## Ethics Statement

The studies involving human participants were reviewed and approved by Ethics Committee for Clinical Research of Verona and Rovigo Provinces. The patients/participants provided their written informed consent to participate in this study.

## Author Contributions

MR, MD, VG, and SM contributed to the conception and design of the study. VG, LM, SM, MA, DL, BI, and BM performed the experiments and analyzed the data. SM developed bioinformatic pipelines for ONT data analysis. AS, AB, MD’A, and GN revised the intellectual and clinical content of the manuscript. MR, VG, and SM wrote the manuscript. MR and MD acquired funding. All authors contributed to manuscript revision, read, and approved the submitted version.

## Funding

The work was supported by the University of Verona Joint Project JPVR18RY8Y. The founding sponsor had no role in the design of the study; in the collection, analyses, or interpretation of data; in the writing of the manuscript; and in the decision to publish the results.

## Conflict of Interest

AS, MD and MR are partners of Genartis srl. The remaining authors declare that the research was conducted in the absence of any commercial or financial relationships that could be construed as a potential conflict of interest.

## Publisher’s Note

All claims expressed in this article are solely those of the authors and do not necessarily represent those of their affiliated organizations, or those of the publisher, the editors and the reviewers. Any product that may be evaluated in this article, or claim that may be made by its manufacturer, is not guaranteed or endorsed by the publisher.

## Footnotes

^1^ https://samplix.com/primer

^2^ https://samplix.com/calculations

^3^ https://github.com/lindenb/jvarkit

^4^ https://github.com/lh3/seqtk

^5^ http://emboss.open-bio.org/rel/dev/apps/cons.html

^6^ https://github.com/nanoporetech/medaka

^7^ https://github.com/MaestSi/CharONT

^8^ https://github.com/MaestSi/MosaicViewer_FMR1

^9^ http://www.bioinformatics.babraham.ac.uk/projects/fastqc/

^10^ https://arxiv.org/abs/1303.3997

^11^ https://ftp-trace.ncbi.nlm.nih.gov/ReferenceSamples/giab/release/NA12878_HG001/latest/GRCh38/supplementaryFiles/HG001_GRCh38_GIAB_highconf_CG-IllFB-IllGATKHC-Ion-10X-SOLID_CHROM1-X_v.3.3.2_annotated.vcf.gz
