# Supplementary material for: Characterization of FMR1 Repeat Expansion and Intragenic Variants by Indirect Sequence Capture
Source: Front Genet. 2021 Sep 27;12:743230. doi: 10.3389/fgene.2021.743230 (PMC8504923; doi:10.3389/fgene.2021.743230)
Supplement: Supplementary file 1 [file Data_Sheet_1.PDF]

SUPPLEMENTARY FIGURES

Characterization of *FMR1* repeat-expansion and intragenic variants by indirect sequence capture

Valentina Grosso[1], Luca Marcolungo[1], Simone Maestri[1], Massimiliano Alfano[1], Denise Lavezzari[1], Barbara Iadarola[1], Alessandro Salviati[1], Barbara Mariotti[2], Annalisa Botta[3], Maria Rosaria D'Apice[4], Giuseppe Novelli[3,5,6], Massimo Delledonne[1,7] and Marzia Rossato[1,7]

[1]Department of Biotechnology, University of Verona, Verona, Italy
[2]Department of Medicine, Section of General Pathology, University of Verona, Verona 37134, Italy
[3]Department of Biomedicine and Prevention, Medical Genetics Section, University of Rome "Tor Vergata", Italy
[4]Laboratory of Medical Genetics, Tor Vergata Hospital, Rome, Italy.
[5]IRCCS Neuromed, Pozzilli (IS), Italy
[6]Department of Pharmacology, School of Medicine, University of Nevada, Reno, NV 89557, USA
[7]Genartis, Verona, Italy

SUPPLEMENTARY FIGURE S1

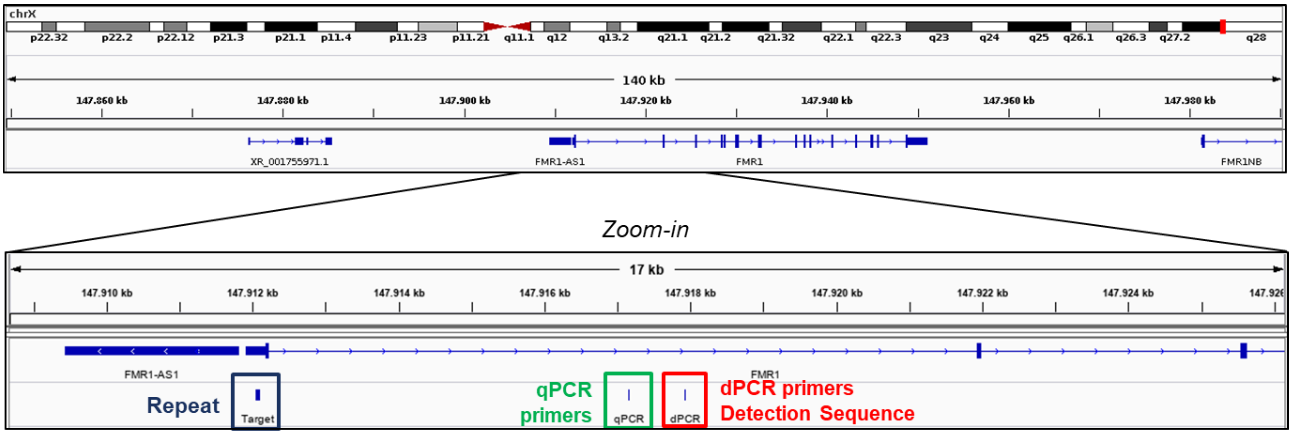

Supplementary Figure S1: Localization of dPCR and qPCR primer pairs at the *FMR1* locus. Integrative Genomics Viewer (IGV) visualization of the *FMR1* locus on the X chromosome (chrX:147,909,919–147,953,125). Zoom-in box shows the localization of the *FMR1* microsatellite in exon 1 (blue), primers used to amplify the detection sequence (DS) by qPCR (red), and to assess *FMR1* enrichment by qPCR (green) after applying the Xdrop workflow.

SUPPLEMENTARY FIGURE S2

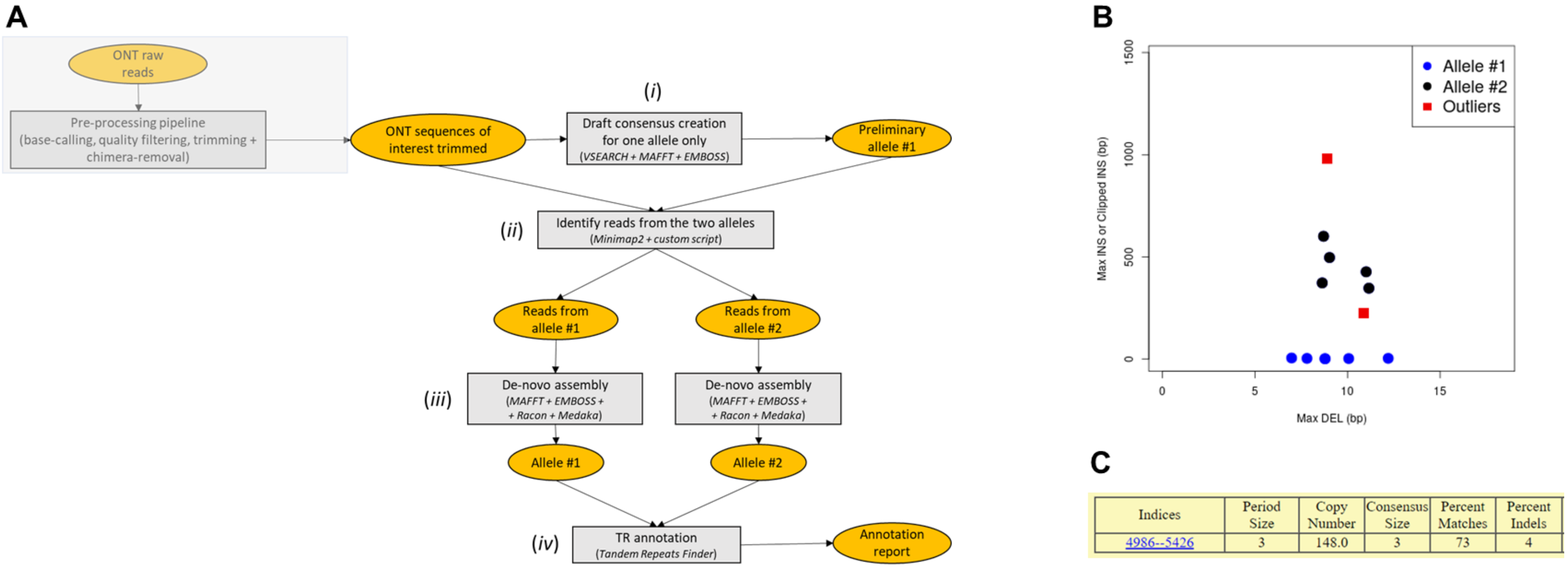

Indices	Period Size	Copy Number	Consensus Size	Percent Matches	Percent Indels
4986--5426	3	148.0	3	73	4

Supplementary Figure S2: CharONT pipeline flowchart and data visualization. (**A**) The CharONT pipeline flowchart. **(i)** ONT trimmed reads that completely span the repeat region are clustered to obtain a preliminary consensus sequence for allele #1. **(ii)** Reads from the two alleles are identified based on *k*-means clustering for **(iii)** subsequent *de novo* assembly. **(iv)** Consensus sequences of the two alleles are searched for repeat motifs. (**B**) Visualization of read clusters, and assignment to the two alleles. (**C**) A representative repeat annotation report.

SUPPLEMENTARY FIGURE S3

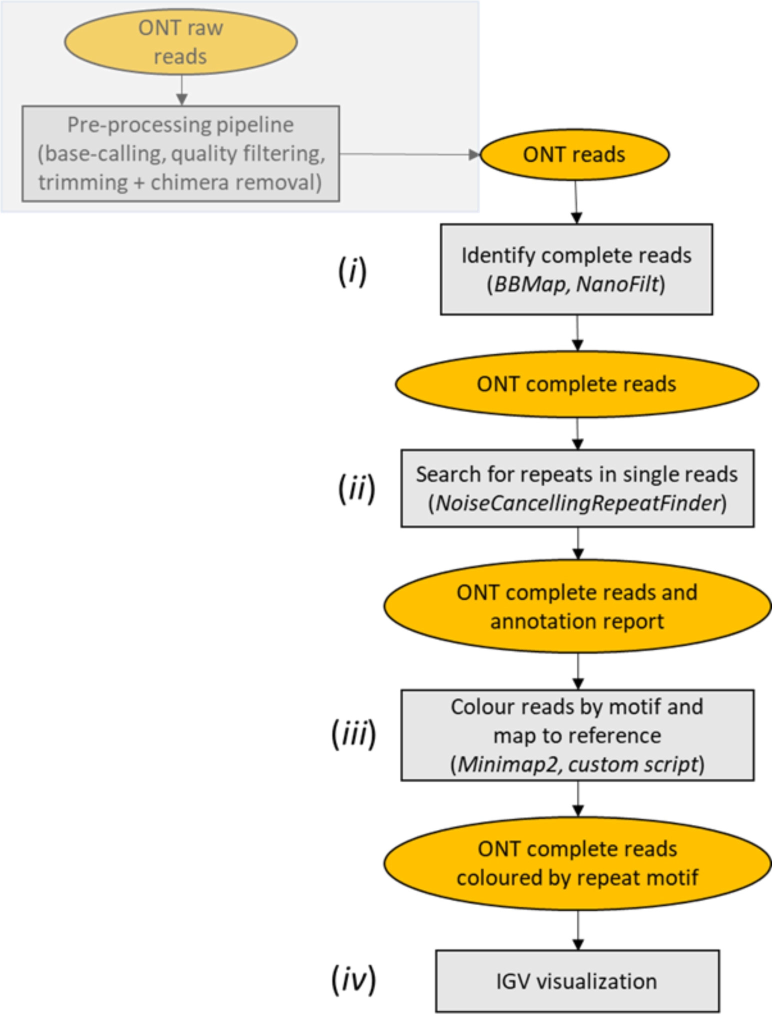

Supplementary Figure S3: MosaicViewer_FMR1 pipeline flowchart. (i) ONT "complete" reads are identified (reads completely spanning the FMR1 repeat region). **(ii)** Complete reads are searched for repeat motifs. **(iii)** A simplified version of the reads can be generated by replacing the sequence with the annotated motif, and both complete reads and complete simplified reads are aligned to the sequence flanking the repeat. **(iv)** Alignments are visualized in IGV genome browser.

SUPPLEMENTARY FIGURE S4

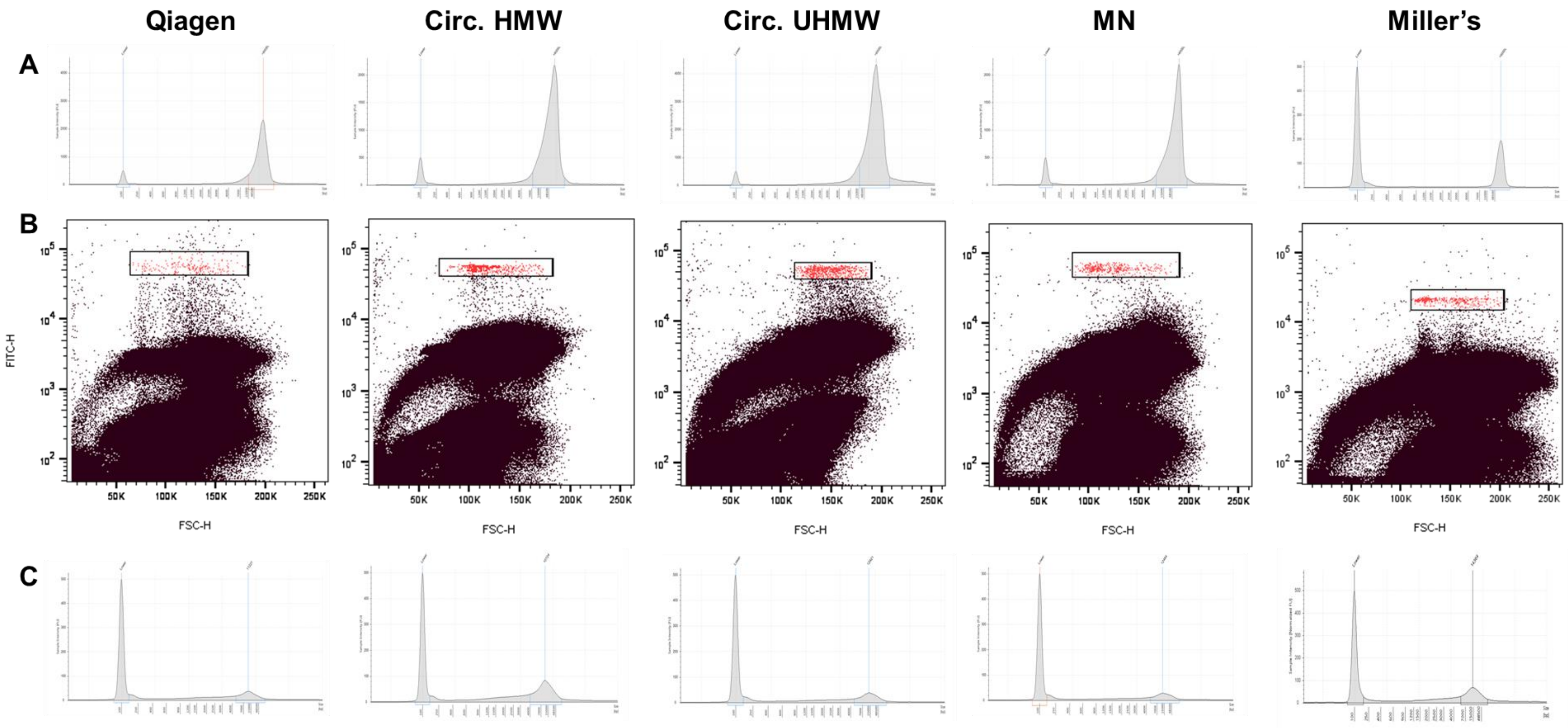

Supplementary Figure S4: Comparison of DNA fragment size and flow-sorting from genomic DNA extracted with different methods. Genomic DNA was extracted with the Genomic Tip kit (Qiagen), Circulomics Nanobind CBB Big DNA Kit using either the HMW protocol (Circ. HMW) or the Ultra-HMW protocol (Circ. UHMW), the NucleoSpin Blood Mini kit (Macherey-Nagel, MN), or Miller's protocol (Coriell samples). (**A**) Fragment distribution of starting genomic DNA samples obtained by capillary electrophoresis. (**B**) FACS dot plots showing forward scatter (FSC-H) *vs* fluorescence intensity (FITC-H) of droplets obtained after the dPCR step. The gate (red events) identifies the positive droplets that are sorted. (**C**) Fragment distribution of dMDA target DNA samples obtained by capillary electrophoresis.
